# Low Risk Meets High Stakes: Unraveling the Mystery of Low D-dimer Pulmonary Embolism

**DOI:** 10.7759/cureus.51045

**Published:** 2023-12-24

**Authors:** Sadat Kasanga, Abdallah Khashan, Ahsan Salik, Ahmed M Aboshehata, Sebastian Casillas, Mohammed Islam

**Affiliations:** 1 Internal Medicine, Raritan Bay Medical Center, Perth Amboy, USA; 2 Internal Medicine, Royal Shrewsbury Hospital, Shrewsbury, GBR; 3 Medicine, Dorrington Medical Associates, Houston, USA

**Keywords:** ct pulmonary angiography, hypoxia, well's score, d-dimer, pulmonary embolism (pe)

## Abstract

Pulmonary embolisms (PEs) are potentially life-threatening emergencies that carry significant morbidity and mortality. Advances in treatment options and the safety of existing procedures have effectively reduced the long-term and short-term effects of the condition. Therefore, it is important to make an early diagnosis so that treatment options can be thoroughly explored. The D-dimer is an important tool in the early diagnosis of PEs. It is especially useful in ruling out the diagnosis in patients with a low to moderate suspicion of the disease. We present a case of a 22-year-old male who presented with exertional dyspnea, congestion, and rhinorrhea for one day and was noted to have persistent hypoxia and tachycardia. The influenza test was positive, and he was started on oseltamivir. Due to persistent hypoxia, a CT pulmonary angiogram was ordered and revealed filling defects in the left lower lobe segmental vessels suggestive of PE, as well as multifocal multilobar bilateral ground-glass opacities. He was initially treated with a heparin drip and subsequently switched to eliquis. After a significant improvement in his hypoxia, he was discharged home for outpatient follow-up, including a hypercoagulable workup. This case demonstrates that despite the usefulness of the D-dimer as a diagnostic tool for PEs, it cannot solely or fully replace the full gamut of screening tools used to determine the risk of PE. Although rare, false-negative scores do occur; therefore, the tool should always be used in conjunction with other scoring systems, physician gestalt, and within the specific clinical context.

## Introduction

Pulmonary embolism (PE) represents a serious medical condition characterized by the obstruction of pulmonary arteries. They are potentially life-threatening emergencies and carry significant morbidity and mortality, especially when misdiagnosed or left untreated [1]. However, diagnosing the condition can be challenging, often requiring a high index of suspicion and the use of multiple clinical and laboratory tools to aid in the decision for further testing and treatment. The challenge in diagnosis often arises from the fact that presenting symptoms can be highly nonspecific and inconsistent, overlapping with various other medical conditions, and therefore, making PE susceptible to being overlooked [2]. Despite this challenge, early detection is essential for mitigating the morbidity and mortality associated with the condition. This highlights the importance of risk stratification tools. Among these, the D-dimer has historically been useful in decision-making, especially as an exclusion tool, given its high sensitivity and negative predictive value [3]. We present a case, however, in which a negative D-dimer was found in a patient who was diagnosed with PE.

## Case presentation

**Figure 1:**
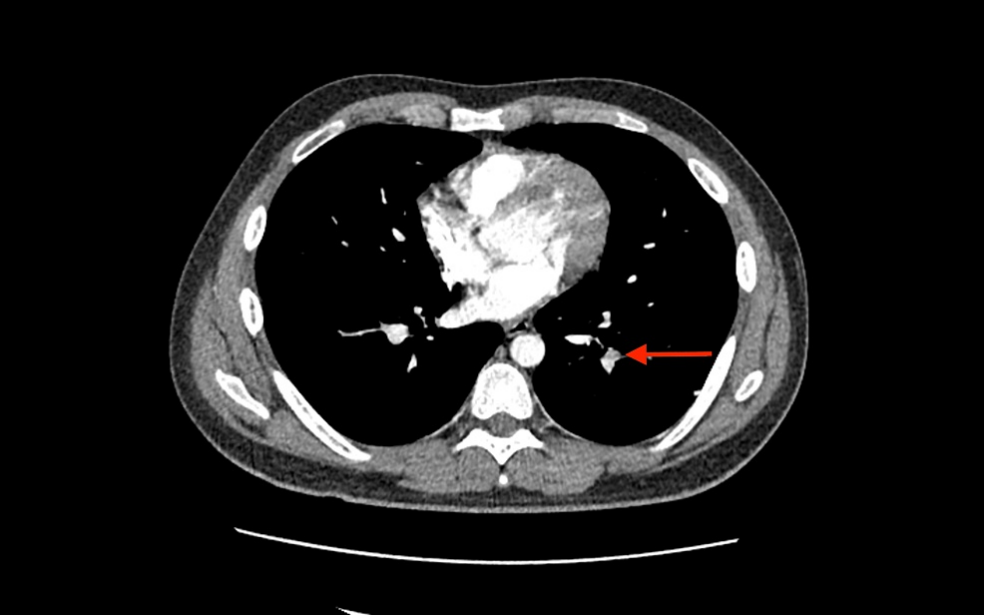
CT pulmonary angiogram demonstrating left lower lobe segmental filling defect (red arrow)

The patient is a 22-year-old male with no significant past medical history or surgical history and no family history of thrombophilia. He presented with exertional dyspnea, congestion, and rhinorrhea of one day's duration following influenza exposure two days prior to the onset of symptoms. The initial examination revealed tachycardia, confirmed to be sinus tachycardia on EKG, and respiratory distress with decreased breath sounds bilaterally. The patient was found to be hypoxic on room air, necessitating nasal cannula oxygen supplementation. Laboratory tests upon admission showed a white blood cell count (WBC) of 12.8, with no abnormalities on the comprehensive metabolic panel (CMP). COVID-19 testing was negative. The WELL's score was initially noted to be 1.5 by the emergency physician and so the patient was initially started on oseltamivir and supportive treatment. However, due to the presence of persistent hypoxia and tachycardia, the clinical suspicion of PE was deemed high enough to warrant ruling out. A D-dimer test ordered earlier returned negative; however, a CT pulmonary angiogram was ordered as well based on the higher suspicion, which revealed filling defects in the left lower lobe segmental vessels suggestive of PE, along with multifocal multilobar bilateral ground-glass opacities (Figure 1).

The patient was initiated on a heparin drip and continued to get supportive care and oseltamivir. He was transitioned to eliquis and subsequently discharged when the hypoxia resolved, with plans for outpatient follow-up and a hypercoagulable workup.

## Discussion

The D-dimer assay stands as a highly sensitive test frequently employed in the evaluation of PE. This test quantifies monoclonal antibodies targeting D-dimer, a byproduct of fibrinolysis, thus reflecting coagulation activity [4]. Initially, D-dimer levels rise during fibrin clot formation, gradually diminishing as clot organization and adherence commence. Notably, the D-dimer test has a relatively brief half-life of four to six hours but remains elevated for about seven days post-clot formation. Nevertheless, a negative D-dimer assay is generally deemed reliable, particularly in low- and moderate-risk patients [4].

Several factors could contribute to the discordance observed in this case. Subsegmental emboli, though smaller in size, can still cause significant clinical symptoms and compromise pulmonary function. However, there exists a correlation between the extent and location of VTE and D-dimer levels [5]. This correlation may potentially explain the lack of a measurable elevated D-dimer level. The clinical significance of subsegmental emboli has been debated, with the 2019 ESC guidelines acknowledging their potential clinical importance and advising tailored management decisions based on the patient's overall condition [6].

Multiple scoring systems have been proposed for risk stratification of patients to determine the need for further testing, including the WELL’s score for PE, the revised Geneva score, the CHOD score, and the Padua score, each with varying predictive levels [7]. As useful as the existing predictive scores are as a tool for guidance, they each have their pitfalls that limit universal application and, therefore, need to be applied in the context of the patient's presentation. Some studies favor the WELL’s score over the revised Geneva score or the simplified revised Geneva score, while others find no significant difference or even advocate for clinical judgment, often termed "physician gestalt," as a superior alternative [8-11]. The findings from meta-analyses generally indicate a lack of consistent distinctions between clinical decision instruments, with some studies suggesting a slight preference for the WELL’s score, or no variance compared to physician gestalt [12-15]. However, it is essential to consider variations among individual clinicians in gestalt performance [16]. Recent research has allayed concerns regarding the WELL’s score's inter-rater reliability, which includes a subjective criterion pertaining to PE's likelihood as a diagnosis [17,18]. As useful as the existing predictive scores are as a tool for guidance, they each have their pitfalls that limit universal application and, therefore, need to be applied within the specific context of the patient's presentation taking into account all available information and tools at the disposal of the physician.

## Conclusions

While D-dimer has proven to be a sensitive marker for detecting fibrinolysis and is often used as a screening tool for PE, it is important to recognize its limitations. D-dimer levels can be influenced by various factors, including age, renal impairment, and comorbidities. False-negative D-dimer results have been reported in patients with localized clot formation, such as subsegmental pulmonary emboli, where the extent of fibrinolysis might not be sufficient to trigger a substantial D-dimer release. This phenomenon raises questions about the appropriateness of relying solely on D-dimer in cases of suspected PE, especially when clinical symptoms and imaging studies suggest otherwise. This case report highlights the multidimensional nature of diagnosing PE. A comprehensive approach, combining clinical assessment, imaging studies, and laboratory findings, is essential for an accurate diagnosis and appropriate management, especially in cases where D-dimer results appear discordant with the clinical picture.
